# Seasonal Patterns in the Frequency of *Candidatus* Liberibacter Asiaticus in Populations of *Diaphorina citri* (Hemiptera: Psyllidae) in Florida

**DOI:** 10.3390/insects14090756

**Published:** 2023-09-11

**Authors:** Timothy A. Ebert, Dalia Shawer, Ron H. Brlansky, Michael E. Rogers

**Affiliations:** 1 Citrus Research and Education Center, University of Florida, 700 Experiment Station Rd., Lake Alfred, FL 33850, USA; rhby@ufl.edu (R.H.B.); mrgrs@ufl.edu (M.E.R.); 2 Department of Economic Entomology, Faculty of Agriculture, Kafr Elsheikh University, Kafr Elsheikh 33516, Egypt; dalia_shamel@yahoo.com

**Keywords:** citrus greening, Asian citrus psyllid, prevalence, group testing, vector ecology, vector–pathogen interactions

## Abstract

**Simple Summary:**

*Candidatus* Liberibacter asiaticus (*C*Las) is a putative causal agent of Huanglongbing, a serious citrus pathogen. *Diaphorina citri* Kuwayama (Hemiptera: Psyllidae) is the primary vector of *C*Las. Florida only has *C*Las, and the only vector of *C*Las in Florida is *D. citri*. We explored patterns in the proportion of psyllids carrying *C*Las by analyzing psyllids collected from seven field sites from 2008 through February 2012. There was an upsurge in prevalence in the fall and, in some groves, a second peak in the spring. We also found that females were more common in the first and last months of the year. *D. citri* has different abdominal colors that we grouped into blue/green and gray/brown. Gray/brown psyllids had a higher *C*Las prevalence than blue/green individuals. We suggest several explanations for these patterns, but the two most likely involve a hypothesized *C*Las gradient in the first few millimeters of new growth and differences in immune response related to sex and color. In application, the results confirm that psyllid management is always necessary because psyllids carry *C*Las year-round. The seven study sites had seven levels of management, from abandoned to intensively managed. In general, more aggressively managed groves had fewer psyllids with *C*Las. A well-managed organic grove was an exception, having low *C*Las prevalence but abundant psyllids. It is possible that the low *C*Las prevalence at this grove was due to management techniques or that this area was infected more recently.

**Abstract:**

*Candidatus* Liberibacter asiaticus (*C*Las) is one of the putative causal agents of huanglongbing, which is a serious disease in citrus production. The pathogen is transmitted by *Diaphorina citri* Kuwayama (Hemiptera: Psyllidae). As an observational study, six groves in central Florida and one grove at the southern tip of Florida were sampled monthly from January 2008 through February 2012 (50 months). The collected psyllids were sorted by sex and abdominal color. Disease prevalence in adults peaked in November, with a minor peak in February. Gray/brown females had the highest prevalence, and blue/green individuals of either sex had the lowest prevalence. *C*Las prevalence in blue/green females was highly correlated with the prevalence in other sexes and colors. Thus, the underlying causes for seasonal fluctuations in prevalence operated in a similar fashion for all psyllids. The pattern was caused by larger nymphs displacing smaller ones from the optimal feeding sites and immunological robustness in different sex–color morphotypes. Alternative hypotheses were also considered. Improving our understanding of biological interactions and how to sample them will improve management decisions. We agree with other authors that psyllid management is critical year-round.

## 1. Introduction

A major threat to world citrus production is a Gram-negative phloem-limited fastidious bacterium, *Candidatus* Liberibacter asiaticus (*C*Las) [1]. *C*Las is one of three putative causal agents for Huanglongbing [2]. *Diaphorina citri* Kuwayama (Hemiptera: Liviidae) is the primary vector of *C*Las, but *Trioza erytreae* Del Guercio (Hemiptera: Triozidae) is also a vector [3]. Management activities are an additional production expense that reduces growers profits. A better understanding of the seasonal changes in the proportion of *D. citri* carrying *C*Las could improve psyllid management practices by targeting times when the risk of transmission was greatest [4]. Changes over time in *C*Las in *D. citri* have been reported [5,6], but we were looking for a seasonal pattern.

Seasonal variability in the abundance of *D. citri* is well studied [7,8,9,10,11,12,13,14,15,16,17]. Often, such studies relate temperature and humidity effects to psyllid population fluctuations. However, the key factors (average temperature, maximum temperature, humidity, and presence of new growth) identified in these studies are inconsistent. The link between temperature and plant development is well documented [18]. The relationship applies to insects like *D. citri* [19,20,21], but there is regional variability in the response [22]. *Diaphorina citri* requires new growth (often called flush) to complete its lifecycle [23]. Thus, seasonal migration occurs at times when there are many flushes [24]. The relationship between the availability of flush and *D. citri* abundance is well documented [25,26,27,28,29]. *D. citri* abundance is linked to the presence of flush, and psyllid development depends on accumulated thermal units. With the seasonal cycle in temperature, it will take longer for psyllids to develop in cooler months and less time in warmer months. This may influence the exposure of these insects to *C*Las.

There is seasonal variability in the abundance of *C*Las in the plant, which has both biotic and abiotic components. From the time of successful infection, *C*Las titers increase to a maximum. By one estimate, the maximum is reached in about 200 days (in greenhouse conditions), after which symptoms develop [30]. However, months or years may pass between infection and symptom development in the field [31,32] and this indicates a slower population growth rate in field conditions. *C*Las titers increase with time spent below 15 °C and cumulative rainfall in the preceding 15 days, while titers decrease with the number of hours above 30 °C [33]. Thus, for a given scion-rootstock, there is a weather effect where warmer and drier areas reduce *C*Las titers, at least for ‘Valencia’ or ‘Natal’ sweet orange on ‘Swingle’ rootstock [33]. There is some effect of rootstock [34,35] and scion [35,36] on *C*Las titers in the leaves. Seasonal cycles in citrus hosts could result in seasonal cycles in the vector if the observed variation is also present in the flush where the nymphs develop.

A prominent feature of *D. citri* is that individuals can be brightly colored. These colors include blue, green, red, orange, brown, gray (nearly white), and yellow. It is easy to categorize extreme individuals, but there are intermediate color forms. Researchers group colors to reduce errors from inconsistently assigning intermediate forms to a specific group: blue/green, gray/brown, orange/red/yellow. Color differences in this psyllid were recorded as early as 1927 [37]. While abdominal color may be indicative of biological status (e.g., mated vs. unmated), laboratory trials showed that adults can change color depending on age, mating status, and food quality [38]. This contrasts with an insect like the melon aphid (*Aphis gossypii* Glover (Hemiptera, Aphididae)), where abdominal color does not change for an individual. Stressed individuals are yellow, and non-stressed individuals are green, though here too there are intermediate forms [39].

Color differences correlate to behavioral, life-table, biochemical, and ecological traits. In *D. citri* blue/green females are more fecund than gray/brown individuals [40]. Mating with gray/brown males results in a higher fecundity on the second day, but there is a higher fecundity by the sixth day for females mated with blue/green males. Female abdominal color does not influence egg fertility, but females mating with blue/green males increased egg fertility over mating with gray/brown males (65% vs. 48%, respectively). While egg fertility declines with female age, the rate of decline is less for females mated to blue/green males. Blue/green females are more attractive to males when given a choice between blue/green and gray/brown females, but the difference is only significant for gray/brown males. The orange/yellow morph has the greatest biomass, followed by blue/green, and gray/brown individuals are the smallest [40,41]. Irrespective of sex, blue-green morphs are more likely (32%) to engage in sustained flight relative to the gray/brown morph (<5%) [42]. This should correlate to the potential risk of long-distance *C*Las movement in the environment. Differences in size associated with sex and abdominal color should have different energy requirements, but the expected differences in feeding behavior are not observed [43,44]. Color morph is related to endosymbiont and *C*Las infection, wherein blue morphs had lower titers of endosymbionts and *C*Las [45], though some disagree [46] and there can be an environmental component [47]. In a separate study that did not track color, males had higher *C*Las titers than females [48]. There are biochemical differences between color morphs that influence insecticide response [49]. That outcome suggests that there should be a similar result for survival on suboptimal or non-host plants [50,51,52], where a morphotype exhibiting elevated insecticide resistance also detoxifies plant secondary compounds more effectively, as shown in other insects [53]. This would provide the ability to utilize a wider range of non-host plants, thereby enhancing survival during long-distance migrations. The color forms of *D. citri* are not just ornamental. Color correlates with the expected fitness of the individual and their ability to vector diseases through the landscape.

The primary goal of this research was to identify a seasonal pattern in the prevalence of *C*Las in *D. citri*. We also wanted to identify factors that might be important in determining *C*Las prevalence in *D. citri*. There are several ways to generate a seasonal pattern. Acquisition rates could be constant but different in subgroups (male vs. female, for example), and seasonal changes in the proportions of subgroups result in an apparent seasonal pattern in *C*Las prevalence. The titers in the insect could be related to age, and there is a seasonal fluctuation in the age distribution. There could be a seasonal component to the physiological susceptibility of the psyllid to *C*Las. There should be a seasonal fluctuation in the proportion of flushes that are *C*Las-free because *C*Las induces off-season flush events [54], and flushing promotes *C*Las movement in the plant [55]. Finally, the acquisition rates could have a seasonal component generated by a seasonal fluctuation in the *C*Las titer in the plant [55].

Our data are estimates of prevalence (a fraction of the psyllid population with *C*Las). Estimates of prevalence are an assessment of the relative risk in an area at a specific point in time. To fully understand the actual risk at a specific location, prevalence must be linked with accurate estimates of vector abundance, migration potential, migration survival probabilities, the titer of the pathogen in the vector, and the number of bacterial cells required to cause an infection in both the host and the vector. Endosymbionts may complicate the relationship as *C*Las–endosymbiont interactions can be antagonistic [48,56,57,58,59], and that would change the number of *C*Las cells necessary to infect *D. citri*. These issues exceeded the scope of the current work.

Considerable time has passed between data collection and publication. We shared sample results with growers shortly after sample analysis. However, the long-term goal was to improve our understanding of factors that influence the biology of this system as a general pattern, recognizing that each site represents a unique treatment combination of infection date, tree age, soil type, pest management, fertilization, irrigation, scion, and rootstock. Finding general patterns that may overarch these unique features requires multiple years of data. Knowing a specific infection rate in one grove on a specific date was not the goal of this research because such information at best helps one grower. Globally, there are several areas where *C*Las has invaded, and through intensive studies at each location, we may learn enough about the biology of this system that we can achieve more favorable outcomes than the current situation [60]. Such information may also enable management practices that improve the longevity of resistant cultivars currently under development.

## 2. Materials and Methods

### 2.1. Simplified Overview

**Figure 1:**
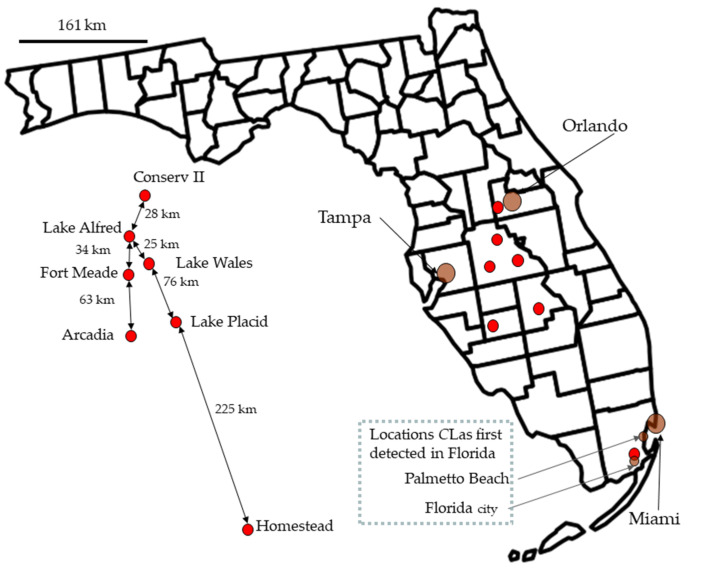
A map of Florida shows the field collection sites and the two sites where *Candidatus* Liberibacter asiaticus was first collected in the state [62]. Names and distances are on the left side of the figure, but the red dots were repositioned to make the text readable.

Monthly collection trips to six regions in the central Florida citrus growing area were undertaken for psyllid collections (Figure 1): Conserv II, Lake Alfred, Lake Wales, Fort Meade, Arcadia, and Lake Placid. Additionally, one sampling site was at the Southern tip of Florida, at Homestead. Samples were collected from January 2008 through February 2012, though not all sites were sampled the entire time. Sampling was by sweep net or aspirating adults off trees. Samples were returned to the lab and sorted by sex and abdominal color. Psyllids within samples were pooled, with 1 to 6 adult psyllids per pool depending on the expected prevalence [61], where prevalence is synonymous with the infection rate (number positive/number tested), and prevalence*100 equals the percentage infected. DNA was extracted from each sample, and PCR was used to detect the presence/absence of CLas. Each plate had three controls: a blank and a known positive, and each well was tested for host DNA using *COX* for plants and *wingless* for insects. PooledInfRate was used to estimate prevalence from pooled samples, and the data were analyzed in SAS. Seasonal variability was modeled using a nonlinear regression with up to six parameters. The best model was selected using AICc. Weather data were downloaded from the Florida Automated Weather Network.

### 2.2. Detailed Methods

#### 2.2.1. Field Sites

In 2008 and 2009, five sites were sampled for psyllids, and a sixth site was added in February 2009. The first five study sites were along the central Florida ridge near the cities of Lake Alfred, Fort Meade, Lake Wales, Lake Placid, and Arcadia. The sixth site was at the University of Florida Tropical Research and Education Center in Homestead, near the Southern tip of Florida (Figure 1). In April 2010, we added Conserv II to our sampling locations.

We classified management styles to describe the study sites. Highly managed groves were ones with an aggressive insecticide program for psyllid control and where the grower removed Huanglongbing-infected trees. Managed groves used insecticides occasionally for psyllid control, and infected trees remained in place. Minimally managed groves were harvested, but spraying, watering, and fertilizing tasks were executed infrequently. Abandoned groves had nothing performed on them except for occasional mowing.

Lake Wales was a highly managed organic grove, but a minimally managed grove was across the road to the west. Psyllids were collected within 3 km of N27.867525 W81.69527833.

Lake Placid: Most samples came from within 5 km of the intersection of Highway 27 and Highway 70 (N27.21383 W81.32883667). These were mostly minimally managed groves. The highly managed neighboring groves contributed a few psyllids due to aggressive psyllid management programs.

Lake Alfred: Initial samples were from highly managed groves belonging to the University of Florida Citrus Research and Education Center. As of October 2008, samples came from two managed groves within 1 km of N28.11520667 W81.74932833.

Arcadia (N27.21041167 W81.67705333) was a grove covering several square miles of highly managed trees with aggressive psyllid scouting, psyllid management, and frequent tree removal. Within this area, several small groves were managed less aggressively. These small groves were less than 30 m from the highly managed trees.

Fort Meade: Collections at this location were primarily from four groves. At one site (N27.72949 W81.69527833), there were a pair of highly managed groves. The other site (N27.795495 W81.744165) had another pair of groves. One of these was highly managed, while the other was minimally managed.

Homestead (N25.50824 W80.50296833): This site was at the University of Florida Tropical Research and Education center. Grove management included irrigation and mowing, but not psyllid control. There were four rows of Persian limes, two rows of Valencia oranges, one row of Ruby Red grapefruit, and one row of Marsh SF-57-4-XE flat Seville grapefruit. All trees showed symptoms of Huanglongbing, but the limes were in better condition with fewer dead limbs and greener foliage.

Conserv II: Early gold sweet oranges were sampled at this location (N28.470435 W81.65316167). The first collection was in April 2010. The area was part of the Water Conserv II distribution center. A cooperative water reuse program by the city of Orlando, Orange County, the agricultural community, and the Mid-Florida Citrus Foundation. The grove was part of the Mid-Florida Citrus Foundation’s A.H. Krezdorn Grove at rapid infiltration basin site 3, an aquifer recharge area.

#### 2.2.2. Psyllid Collection

The sampling goal was 600 psyllids per month per site, but abundance was variable. At high densities, we focused on collecting equal numbers from each of the 20 trees. At low densities, we continued sampling until we collected psyllids from at least twenty trees. We tried alternate groves suggested by the owner of the main site if the abundance was too low, but kept the samples for each site separate. Psyllids were collected by sweeping the net, checking the net after sweeping one to five trees, and doing this at least twenty times. Sweeping was from the top of the tree towards the bottom and back up, then moving around the tree, continuing this pattern. This differs from other approaches using the sweep net [63,64]. Fruit, twigs, flower petals, and snails caused problems in collecting psyllids, and frequently checking the net was advantageous. Fruit in the net was used as a sign of excessive force. This approach ensured that each sample was representative of a location and minimally skewed by the status of an individual tree within that grove. Considering that psyllids often prefer field edges [4,65,66], these locations were often searched first, though other locations were included. We used the sweep net method because it enabled the collection of a sufficient sample in a few hours, even at low abundances. The short collection interval makes the samples point estimates rather than averages over time, as one finds with trapping methods like sticky cards. The sweep net method used in this way samples a larger proportion of the tree canopy relative to a method like tap sampling. Sweep net sampling also allowed for a readjustment of trapping effort based on current abundances, which were unknown at the start of collecting each sample. If the foliage was wet, the sample was collected by aspirating psyllids directly from the foliage.

Psyllids were alive when transported to the lab, then stored in 80% ethanol in a refrigerator until they could be sexed. In 2009 and later, samples were both sexed and sorted into blue/green or gray/brown-colored individuals. Psyllids could be bright green or have just a trace of green that was noticeable only in contrast with a gray psyllid. Any trace of green was sufficient to put a psyllid into the green category. Red, orange, yellow, and white individuals were also present, but it was difficult to cleanly separate them from brown/gray. This was because there was a continuous gradation in color. Therefore, all the psyllids were categorized as blue/green or gray/brown, where the gray/brown category also contained the other colors when present. The long-term storage of psyllids and extracted DNA was in a −10 °C freezer. Psyllids in samples with a low expected prevalence rate were pooled before DNA extraction. If the expected prevalence rate was close to zero, up to six psyllids were combined to make a sample. Other studies have pooled up to ten [67]. Pool size decreased at prevalence rates above 5%. Additionally, there was a goal of having at least 20 samples, and pool size was decreased if pooling resulted in fewer than 20 samples.

#### 2.2.3. Analysis for *C*Las

Psyllid DNA was extracted using Qiagen DNeasy blood and tissue kits (Qiagen.com (accessed on 8 September 2023) catalog 69506, (Qiagen, Germantown, MD, USA). The kit was designed for 25 mg of tissue, but an individual psyllid weighs about 0.5 mg. Therefore, we reduced the buffer in the final elution step to 100 μL.

Each 96-well plate had two wells with only Taq, probes, and primers, and two wells contained a positive control. The remaining 92 wells were filled with unknowns, and each unknown was tested twice. The unknown was retested in the event that one well tested positive and the other well tested negative. The sample was assumed to be negative if the retest also had a mixed outcome.

The detection of *C*Las in plant samples has been described elsewhere [68,69]. Detection of *C*las in psyllids was conducted as previously described [68], except that only 8.4 μL of TaqMan Universal PCR Mastermix (Applied Biosystems: ThermoFisher Scientific Inc., Waltham, MS, USA) was used, and primer concentration was 353 nM while probe concentration was 176 nM in a total reaction volume of 17 μL. The *COX* gene was used as an internal control for plant samples [69], and *wingless* was used as the insect internal control [68]. Plates where any of the controls failed were retested.

Examination of 1240 plates run from 2008 through 2012 revealed 29 possible false positives. The estimated error rate was 2.3%, with an upper 95% confidence interval of 3.297%. However, we ran paired wells for each sample. If the true error rate was 3.297%, then the probability of obtaining two false positives would be one in 909 (0.11%). However, this value is a combination of the probability of autodetection and cross-contamination. Plates were retested if there were problems. Two problems could occur. The negative control wells could test positive, or the positive control could test negative for either CLas or host DNA. Two reactions per sample minimized the risk of false positives, and considering all CT values below 40 as positive minimized the risk of false negatives.

#### 2.2.4. Weather

**Figure 2:**
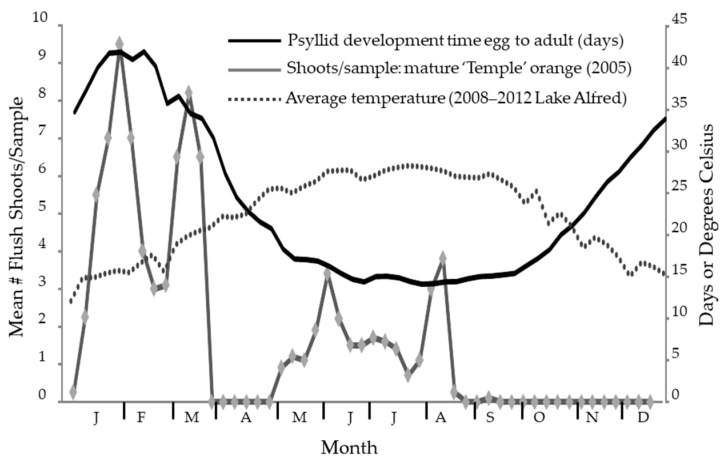
The seasonal fluctuation in temperature at Lake Alfred is plotted superimposed over the estimated development time for *Diaphorina citri* Kuwayama based on the accumulated temperature with the same numeric scale on the right-side y-axis. As shown, an adult that emerges in mid-January must have started as an egg 40 days prior. Periods of flush availability are shown for ‘Temple’ tangor from 2005 data [107].

Weather data were from the Florida Automated Weather Network [70] for Arcadia, Homestead, Lake Alfred, and Avalon Rd (=Conserv II), Frostproof, and Sebring (Table 1). Weather was recorded at 15 min intervals for all years. These stations measure temperature at three heights (60 cm, 2 m, and 10 m), soil temperature (10 cm), relative humidity, solar radiation, rainfall, wind speed, and direction. For each collection date, we described the weather during the period relevant to the developmental period of the psyllids collected on that date. In summer, a psyllid will go from egg to adult in about 15 days, while it may take 60 days in winter (Figure 2), based on a psyllid requiring 253 growing degree days above 10.5 °C to complete development. To make the period of measurement more relevant to psyllid biology, we used a growing degree day model to calculate the date an egg would have been laid to become an adult on our sampling date. We then calculated the average minimum daily values, average maximum daily values, average, and median for each weather variable. Thermal units are commonly used to adjust for yearly differences in weather [29,71], though typically a fixed interval is used.

#### 2.2.5. Data Analysis

There are three binary state variables: sex, abdominal color, and the presence of the bacterium. The proportion of individuals in one state (M vs. F), (blue/green vs. gray/brown), and (*C*Las+ vs. *C*Las−) was estimated using the Excel add-in PooledInfRate [72]. Previously, PooledInfRate and the pooling methodology were examined for suitability in this project [61]. The PooledInfRate methods are also available in R [73]. PooledInfRate is used as part of CDC tracking programs for the West Nile virus [74]. Group testing was developed in 1943 [75] and further improved in many subsequent papers, a few of which are cited herein [61,76,77,78,79,80,81,82,83,84,85,86,87,88,89,90,91,92,93,94,95,96,97,98,99,100,101,102]. Finally, the pooled sample approach has been recommended for use by citrus growers in scouting for *C*Las in the early stages of invasion [103]. In the current work, the pool size for all samples was six or fewer, the exact number chosen based on prior results or after a preliminary test of ten individuals. In the latter case, single and pooled samples were analyzed to give a single point estimate following procedures for the analysis of groups of different sizes [79]. After estimating the infection rate, regression and correlation analyses were conducted using SAS^®^ software (version 7.15 HF9) [104].

Our approach to modeling seasonal variability used nonlinear regression to model a cosine function [105]. There were seven models to choose from in this approach: In the models below, P is prevalence, t is time, τ is the duration of one cycle, β are estimable parameters, and an error term ε. Three-parameter model: P = β_0_ + β_1_t + ε, this model assumes that there is no seasonal trend in these data. Four-parameter model: P = β_0_ + β_1_ cos ((2π/τ)/t + β_2_) + ε, this model assumes that there is a seasonal trend but that it is constant from year to year. Five-parameter model: β_0_ + β_1_ cos((2π/τ)/t + β_2_) + β_3_t + ε, this model assumes that there is year-to-year variability but that the change is linear. Six-parameter model: β_0_ + β_1_ cos((2π/τ)/t + β_2_) + β_3_t + β_4_t^2^ + ε, this model assumes that there is year-to-year variability and allows for both upwards and downwards changes in prevalence from year to year. One must also decide how many cycles are present in a year. If there is only one cycle, then τ = 1. If there were two cycles, then τ = 0.5. The best model was chosen using Akaike weights (ωi), calculated using the corrected Akaike information statistic (AICc) to calculate AIC differences (Δi) [106]. The calculations were completed in Excel based on formula 2.9.1 in the cited work. This was performed for all the data combined as well as for individual groves.

We then looked for correlations between prevalence and the weather variables previously described, along with some biotic variables. The categorical biological variables are sex and color. The continuous biotic variables are percentage male, percentage green male of males, and percentage green female of females. Percentages were arcsine square root transformed prior to analysis. We used the residuals from the seasonality model and a stepwise regression approach (0.049 to enter, 0.05 to stay) to identify significant trends.

We present two approaches to data analysis. In one approach, we used PooledInfRate to calculate 95% confidence intervals (by month, for instance), and significant differences were identified when the confidence intervals did not overlap. In this approach, an individual insect had the same value as any other insect in terms of estimating prevalence. Alternatively, PooledInfRate was used to calculate prevalence for each sample. These estimates were analyzed using ANOVA, and the least squares means are reported. In this approach, samples with fewer than 20 insects were dropped from the analysis to reduce the influence of poor-quality samples. In the latter method, the contribution of an insect to a large sample is proportionately less than that of an insect in a small sample.

## 3. Results

Modeling psyllid developmental times over seasons showed that it takes slightly over 40 days to go from egg to adult during the cooler period at the end of January and beginning of February. As warm temperatures prevail during June, July, August, and September, the development time decreases to 14 or 15 days (Figure 2). Adults can be found at all times of the year in Florida, though the psyllid abundance is lower during the cooler months due to the relative scarcity of the new growth that is necessary for the reproductive cycle of *D. citri* [23]. Psyllids in late summer must survive a period where flushing is scarce to reproduce the following year.

**Figure 3:**
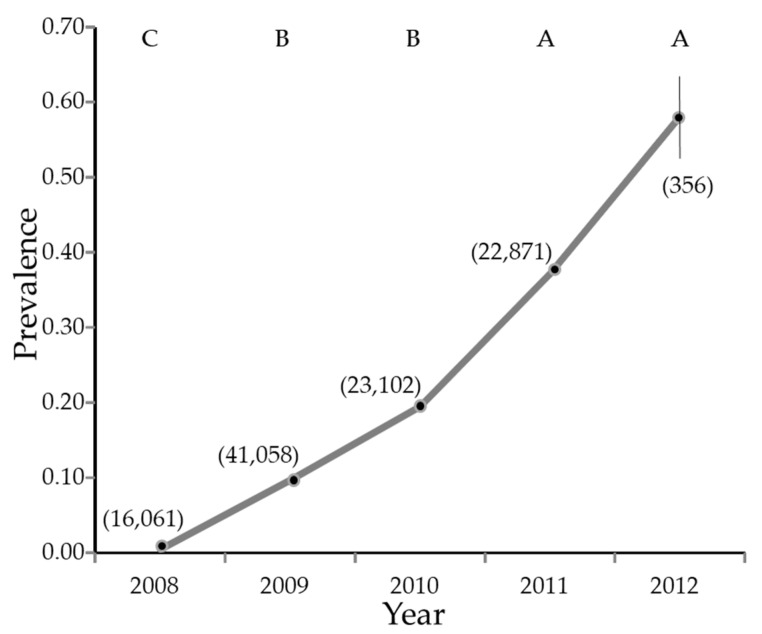
Estimate of percentage of *Diaphorina citri* Kuwayama carrying *Candidatus* Liberibacter asiaticus by year. The vertical line is the 95% confidence interval. Where the line appears absent, the 95% confidence interval is smaller than the symbol used to plot the figure. The numbers in parentheses are sample sizes. Years with different letters have significantly different least squares means (not shown) by Tukey test.

Overall, 129,798 psyllids were collected from January 2008 through February 2012, of which 72,579 were female and 57,219 were male (55.9% female, 95% CI 55.6 to 56.2%). Of these, 103,934 were tested for the presence of *C*Las. The infection rate increased from nearly zero in 2008 to nearly 60% in the first part of 2012 (Figure 3).

Over the entire study, Lake Wales had the lowest incidence, with 0.098% of the psyllids carrying *C*Las (95% CI 0.066 to 0.139%), followed by Arcadia at 1.08% (95% CI 0.89 to 1.30%), Fort Meade at 2.01% (95% CI 1.77 to 2.27%), Lake Placid at 2.80% (95% CI 2.48 to 3.15%), Lake Alfred at 39.36% (95% CI 38.72 to 40.00%), and finally Conserv II at 44.20% (95% CI 43.25 to 45.14%). The incidence at Homestead was 42.47% for psyllids from orange + grapefruit (95% CI 40.26 to 44.70%) and 35.57% for psyllids from lime (95% CI 34.62 to 36.53%). These roughly fall into two groups: high infection rates at Homestead, Lake Alfred, and Conserv II (above 30%) vs. lower infection rates at the other sites (under 5%).

**Figure 4:**
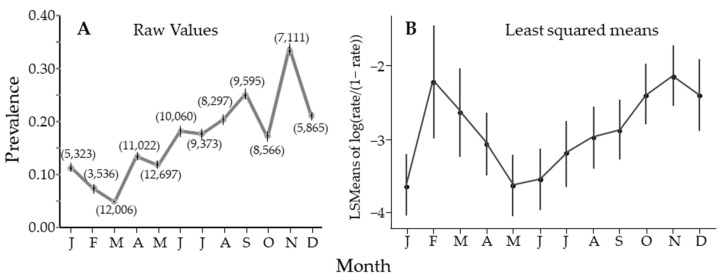
Estimate of percentage of *Diaphorina citri* Kuwayama carrying *Candidatus* Liberibacter asiaticus by month, averaged over years. (**A**) using PooledInfRate to estimate prevalence from all the data for that month. The 95% CI looks like it is missing if the interval is smaller than the symbol used to plot the figure. Numbers in parentheses are sample sizes. (**B**) A similar figure as in (**A**), however, prevalence for each sample was calculated, and the figure is the mean and 95% confidence interval for those data.

Over the entire study, there was seasonal variability, with significant differences between May and August when compared to November, when prevalence peaked (Figure 4A). Figure 4A estimates prevalence using PooledInfRate, where all the samples from all groves and all years for each month were treated as one sample (sample size in parentheses). All months were significantly different from all others, with a single peak in November. Alternatively (Figure 4B), prevalence was estimated for each sample using PooledInfRate, and the individual prevalence estimates were used to build a regression model that showed two peaks. This observation could be seen in the nonlinear regression results, but four of the seven sites had one seasonal peak (Table 2). Either way, there was periodicity in the seasonal fluctuation in the prevalence of *C*Las in *D. citri*, with a clear peak in November and a possible second peak in February.

The residuals from the seasonal model were used to look for other factors that influence prevalence. Two models were examined. The model using dates in a year-month format showed significant effects of abdominal color as well as year, month, and an interaction between the year and month. Alternatively, the Julian date could be used, and the percentage of males with green abdomens and the solar radiation at 2 m were significant predictors (Table 3). Recoding sex and color into a single variable showed a significant effect of color but not sex (Table 4). We note that the correlation in prevalence between these four groups exceeds 78% (Table 5).

**Figure 5:**
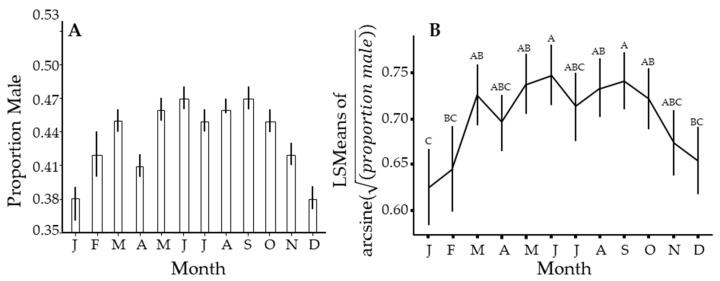
Seasonal changes in the proportion of male *Diaphorina citri* Kuwayama captured over the entire study. (**A**) uses PooledInfRate on all samples for that month. (**B**) is similar but uses PooledInfRate to estimate the proportion for each sample, from which means, confidence intervals, and significant differences by Tukey test were estimated. Months with different letters have significantly different least squares means (not shown) by Tukey test.

There was a seasonal pattern in the sex ratio of this insect, wherein the proportion of males in the population declines from about October through January of the following year to a low of about 38% (Figure 5). This cycle roughly follows the flushing pattern (Figure 2), where a lack of new growth results in a few new individuals being added to the population over this time interval. It appears to track with a dip in the proportion of males in April and a reduced flush during this time, but another dip in July is not easily explained. The proportion of males in the population peaks broadly from April through September, when males were slightly less than half the population.

**Figure 6:**
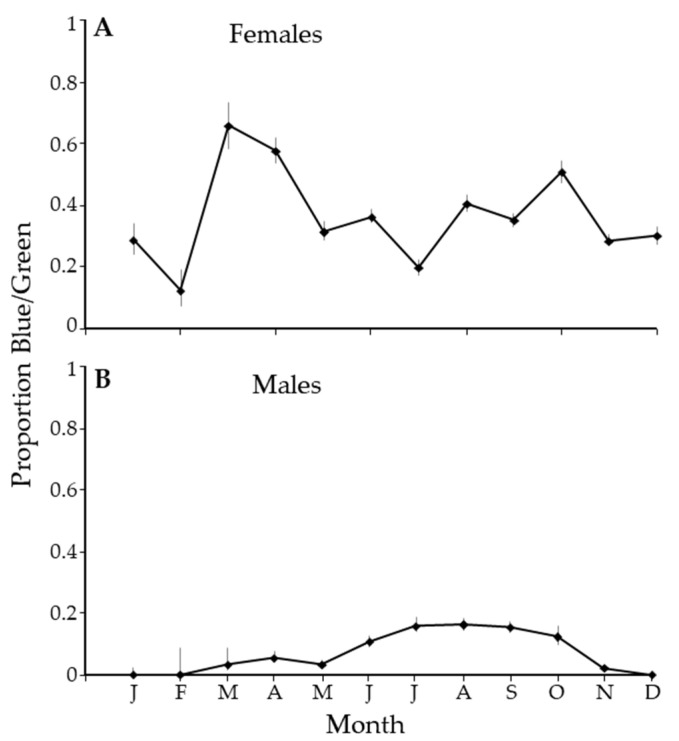
The proportion of blue/green *Diaphorina citri* Kuwayama by month for females (**A**) and males (**B**).

There was an unusual pattern in the ratio of the blue/green morph to the gray/brown morph. In February, there were about 10% of females that were green. The proportion spiked in March with the abundance of new growth, with over 60% of the females being blue/green. Through most of the year, about 40% of the population of females was blue/green (Figure 6A). In contrast, in the winter months, there were no blue/green male psyllids, and the proportion of blue/green males averaged about 20% in the summer (Figure 6B).

## 4. Discussion

We showed a seasonal pattern in the prevalence of *C*Las in the vector *D. citri*, with a clear peak in November and sometimes a second peak in February. However, *C*Las is always present in some fraction of the vector population at all times of the year in Florida. Others report that migration and large numbers of psyllids can occur at all times of the year [108]. While the risk of transmission changes throughout the year, there is no safe period. We showed a long-term trend of increasing prevalence year over year and significant grove differences between our study sites. We showed gray/brown psyllids were more infected than blue/green psyllids, and that agrees with previously published work [45]. There was a consistent difference between the sexes, with females having a higher prevalence, but the difference was not significant in these data. We showed that changes in *C*Las prevalence in each sex–color combination were positively and highly correlated. We showed a seasonal trend in the proportion of males in the Florida population, where samples were more female-dominant in December and January and nearly 50:50 from May through September. Finally, we showed that the proportion of green males tended to be less than the proportion of green females and that green males were absent roughly from December through February. In management terms, these outcomes would support sampling at all times of year because there is a risk of disease progression through the grove and between groves at all times of year. Scientific studies should include both sexes and all colors in the experimental design and analysis. Scouting for grove management can ignore sex and color differences because all psyllids in all seasons may be vectors. The requirements for constant scouting may be relaxed if management goals change from prevention to mitigation and economic injury levels become relevant [109,110].

With all trees at Homestead infected, it was curious why a considerable proportion of the population was PCR-negative for *C*Las. Groves at Lake Alfred also looked 100% infected, but we continued to recover PCR-negative psyllids. We suggest that healthy individuals result from several features of this system: first, healthy psyllids can be in a sample due to migration from healthy branches within or outside of the grove. It takes many years for *C*Las to become systemic in the plant and to infect every tree in the grove. Second, healthy psyllids can arise through different infection dates or microclimates that cause individual trees to have various levels of disease progression. *C*Las titers increase over time from the initial infection [30], but the unequal distribution of *C*Las within the plant allows *C*Las-free tissue on infected plants [111,112,113,114]. The unequal distribution extends to the cellular level [115,116,117], and flush can have remarkably few *C*Las cells [116]. Third, psyllid feeding is not constant. Estimates for adults indicate that less than 30% of the time is occupied with ingesting phloem [118,119,120] when on flush, though 4th or 5th instars spent 66–72% of their time ingesting phloem [119,120]. The time spent ingesting phloem multiplied by the flow rate and the bacterial concentration in the phloem determines the exposure risk of the psyllid. The number of bacterial cells per volume of phloem is further modified by considering the size of the food canal as a filter with a pore size of 460 to 818 nm for first instar or adult psyllids, respectively [121] (p. 11). A bacterial cell ranging in size from 140 to 1500 nm [117,122,123,124] must pass through the food canal for the psyllid to acquire *C*Las [121,125]. Based on these measurements, not all bacterial cells will fit through the food canal. The exposure risk for the plant is modified by the ability of *C*Las to pass through the 380 nm adult salivary canal [121]. Nymphs may acquire *C*Las by ingesting bacteria deposited by adults. The bacterial load delivered at the time of infection of new shoots will influence the acquisition risk of psyllid nymphs that develop on that flush, and uninfected individuals may arise from insufficient time between infection and offspring migration. Assuming an infected female transmits *C*Las to a healthy shoot, the eggs she lays at the time of feeding may become adults 14 days later. However, bacterial titers decline in the first 10–12 days of that period [126]. The first few adults from this shoot may be healthy, even if the other steps in this process were successful. We also do not know the probability of a successful infection given that only 1, 2,…, *n* bacterial cells move into a healthy host plant or vector. A successful infection probably requires several *C*las cells [126], and the number of available cells could depend on scion and rootstock variation in *C*Las titer [35,127,128] though differences are not always observed [129]. Finally, there is a behavioral component where adults on mature leaves are xylem feeders, not phloem feeders [118], and *C*Las is a phloem-limited organism. Therefore, migrating healthy adults will stay healthy longer if they remain on mature leaves. The psyllid is a phloem feeder when on flush and therefore more likely to ingest *C*Las at that time. For these reasons, not all psyllids on *C*Las-positive plants carry *C*Las, and not all plants will become infected when fed upon by a *C*Las-positive *D. citri* [32].

Methodological issues associated with sampling may produce false negatives. If one collects a psyllid that recently acquired *C*Las, then it is likely that the psyllid will have few bacterial cells. Theoretically, the PCR test for *C*Las can detect a single bacterial cell. Assuming the target DNA makes it through extraction and cleaning, testing consists of resuspending the DNA in 100 μL of buffer and adding 1 or 2 μL of the suspension to the PCR reagents. Thus, the probability of detecting a few copies of target DNA in a sample is low; multiple tests on this sample could produce conflicting results. We retested samples when we got contradictory results, thereby minimizing the risk of false negatives. Some sampling methods may add another complication when samples remain in the field for extended periods. Sticky cards are an often-used monitoring tool, but the risk of a false-negative PCR test increases as the DNA degrades [130].

We suggested several explanations for the observed seasonal periodicity in *C*Las prevalence in *D. citri*. The first was a constant infection rate where the proportions of different subgroups in the population changed seasonally. We showed a seasonal change for both sex (Figure 5) and color (Figure 6). However, the pattern was different from that for *C*Las prevalence (Figure 4A or B), where the peak was sharper and displaced relative to Figure 5 and Figure 6. Also, prevalence was highly correlated (>0.79) in all subgroups (Table 5), which suggests that a common process is influencing all groups. The second was a seasonal change in the population age structure. Given that females live longer than males [21,40,131], the sex ratio should change as males die off during periods where reproduction is unlikely. Thus, the age structure of the population becomes older when the population is more female-dominated in January and December (Figure 5). However, from May through October, the ratio is roughly 47:53 (M:F) and indicates a stable age distribution for adults. This stability is inconsistent with the more constant change in *C*Las prevalence in the psyllid (Figure 4). The third was a seasonal change in the susceptibility of the psyllid due to environmental stress. While it is difficult to reconcile the gradual changes in Figure 2 with those in Figure 4, the relationship between weather and physiological stress may be complex [132,133,134]. Fourth, a seasonal pattern could arise through enhanced off-season flushing caused by *C*Las. However, we do not see that the peaks in Figure 1 result in lows in Figure 4. The remaining explanation is that seasonal changes in *C*Las prevalence in *D. citri* result from a seasonal fluctuation in *C*Las titers in the plant at the apical meristem of flush, where psyllid nymphs develop and acquire *C*Las. Our data were highly variable from sample to sample. Likely, all the factors discussed play a role in determining the number of infected psyllids at different spatiotemporal scales and could explain finer-scale variability.

There are several explanations for why there are differences in prevalence correlated with color differences. *C*Las consumes the physiological resources of the psyllid [135]. This additional stress increases the probability that a psyllid will be smaller and gray/brown. However, if *C*Las converts individuals from blue/green to gray/brown, then the pool of infected gray/brown individuals will grow faster than it would otherwise. This would result in different correlations between some groups that are inconsistent with Table 5. *C*Las reduces host quality, and poor-quality hosts should produce more gray/brown individuals. As expected, groves with low *C*Las prevalence in the psyllid had a higher proportion of blue/green morphs. Lake Wales had a low *C*Las prevalence, and the percentage of blue/green psyllids was 31% (95% CI: 31–32%). Homestead had a high prevalence and had 26% blue/green (95% CI 25 to 27%). The percentage of blue/green individuals at Lake Alfred was 23% (95% CI 22 to 23), and the percentage of blue/green individuals at Conserv II was 17% (95% CI 16 to 17%). With this outcome, seasonal fluctuations in flush quality [136] should produce more blue/green individuals when flush is healthier. This could explain the November peak (Figure 4B) when only off-season flush is available (Figure 2), but it does not account for the February peak when flush should be abundant. Two other options are suggested, but our data cannot distinguish between them. First, there is a *C*Las gradient for some distance below the growing point. The gradient exists because the phloem needs to develop, and there must be time for *C*Las to invade the new tissue and replicate. Larger nymphs will push smaller nymphs to less favorable feeding sites, resulting in gray/brown individuals that are more exposed to *C*Las. Psyllid size is correlated sex and color where blue/green individuals are larger than gray/brown and within each color females are larger than males [38]. Second, the immune system of the blue/green morphs is stronger [45,137], and more bacterial cells must be ingested to result in a successful infection. There are also immune response differences between males and females [138], though it is less clear if these would alter prevalence. All these explanations likely occur in the field, but based on our data, we suggest that the latter two had the strongest influence.

*C*Las was first detected at Arcadia in February 2006 [62]. Arcadia lies between the Homestead site, where the first record came from Florida, and study sites in Polk County [64]. Based on proximity, *C*Las has been at Arcadia longer than locations at Fort Meade, Lake Wales, and Lake Alfred (all three locations are further north from the original detection locations (Figure 1)). Consequently, the low frequency of *C*Las at Arcadia would be consistent with the idea that an aggressive management strategy can reduce the proportion of infected psyllids and the rate at which the disease spreads [139,140,141,142,143,144]. An alternative narrative is that human movement resulted in a more rapid spread of *C*Las to more Northern sites. We point out that the detection in Arcadia was only six months after the first positive detection [62]. There is a long asymptomatic phase in disease progression in the plant during which the psyllid can acquire *C*Las. The movement of asymptomatic nursery plants generates many epicenters. Homeowners buy these plants and spread inoculum sources. Infected psyllids rapidly spread the disease into neighboring groves [62]. Under relatively uniform disease pressure, a management influence on prevalence is the simplest explanation. A third scenario is that a combination of slow natural spread and irregular movement of *C*Las through human activities results in a random patchwork of infected and relatively healthy groves. We propose this condition as one possibility for low prevalence at Lake Wales, despite the presence of a Walmart Supercenter with plants for sale less than two miles away. This happening at Lake Wales and in the highly managed groves is less plausible. Low prevalence at Lake Wales may be due to organic management practices creating a unique microenvironmental effect (as generally suggested here [145]), or the grove being infected later.

We showed that there is a seasonal component to the number of psyllids carrying *C*Las. We sampled groves in Florida at monthly intervals. At that scale, there was a peak infection level in November and another peak in February. Not all groves had their February peak. We evaluated several ways of generating a seasonal cycle. The simplest explanation is that it reflects seasonal changes in *C*Las titer in the plant. This explanation would affect all subpopulations equally. There was a high correlation in prevalence between males vs. females and blue/green vs. gray/brown subpopulations. However, the average infection rates were not equal in the subpopulations. This inequality is probably due to insect health and competition between nymphs. Psyllid health may also contribute to the observed seasonal fluctuation in *C*Las prevalence in psyllids. The other explanations did not appear to fit the seasonal pattern. Grove management approaches influenced *C*Las prevalence of psyllid. More aggressively managed groves had a lower prevalence. The number of infected trees changes *C*Las prevalence in the psyllids. However, the trees yielding infected psyllids accomplish this at different rates in different seasons. This cycle is not strong enough in Florida to generate disease-free periods. Therefore, management efforts are a year-round task.

## Figures and Tables

**Table 1:** Weather station and location information.

Study Site Name	Station Name	Station ID	Latitude (deg)	Longitude (deg)	Elevation (ft)
Arcadia	Arcadia	490	N 27.22621	W 81.83838	64
Conserv II	Avalon	304	N 28.47485	W 81.65300	196
Homestead	Homestead	440	N 25.5126	W 80.5031	8
Lake Alfred	Lake Alfred	330	N 28.10185	W 81.71128	154
Ft. Meade	Ona	380	N 27.39750	W 81.93973	75
Lake Wales	Frostproof *	390	N 27.76551	W 81.53735	164
Lake Placid	Sebring	470	N 27.42108	W 81.40095	117

* This site was removed from FAWN in 2021, but the data are still available.

**Table 2:** Comparison of different models to predict seasonal fluctuations in prevalence of *Candidatus* Liberibacter asiaticus in *Diaphorina citri* Kuwayama for all sample sites in Florida and for individual sites.

Site	N	Cycles/Year	k	R^2^
All	967	2	5	0.25
Conserv II	141	1	6	0.50
Lake Alfred	214	1	5	0.65
Arcadia	116	1	6	0.13
Homestead	143	2	6	0.40
Lake Wales	184	2	5	0.17
Lake Placid	61	1	5	0.63
Fort Meade	108	2	6	0.56

**Table 3:** Estimating the infection rate of *Candidatus* Liberibacter asiaticus in *Diaphorina citri* Kuwayama with models based on either categorical or continuous variables. To deal with leap years, we used Julian date divided by days in the year to obtain JulianFrac. NM2Solar is the solar radiation at 2 m. PerMG is the percentage of green males.

Categorical	DF	SS	F	P > F	Source	DF	SS	F	P > F
Model	42	104.82	34.63	<0.01	location	6	72.10	166.73	<0.01
Error	658	47.42			color	1	1.46	20.22	<0.01
Total	700	152.24	R^2^=	0.69	year	3	5.01	23.17	<0.01
					month	11	4.84	6.11	<0.01
					year × month	21	3.83	2.53	<0.01
Continuous									
Model	9	100.27	137.98	<0.01	location	6	73.60	151.92	<0.01
Error	715	57.73			JulianFrac	1	6.30	78.04	<0.01
Total	724	158.00	R^2^=	0.63	NM2Solar	1	2.07	25.61	<0.01
					PerMG	1	0.80	9.97	<0.01

**Table 4:** Estimating infection rate of *Candidatus* Liberibacter asiaticus in *Diaphorina citri* Kuwayama with the degrees of freedom (df) and type III sums of squares (SS III). Location is the sample collection site. SandC is sex and color with four states: brown female, brown male, green female, and green male. Year is from 2008 through 2012. Removing any variable from the full model resulted in a larger fit statistic (AICC, BIC, and HQIC).

	Df	SS III	*F*	P > *F*	Source	df	SS III	*F*	P > *F*
Model	23	101.10	58.19	<0.0001	Location	6	72.04	166.46	<0.0001
Error	677	51.14			SandC	3	1.57	7.23	<0.0001
Corrected Total	700	152.24	R^2^=	0.66	Year	3	5.02	23.20	<0.0001
					month	11	4.81	6.06	<0.0001
					year × month	20	3.82	2.53	0.0002
	Raw Mean %								
Brown female	35.4	A							
Brown male	33.2	AB							
Green female	28.2	BC							
Green male	25.4	C							

**Table 5:** Pearson correlation coefficients for prevalence rates between different sex and color forms of *Diaphorina citri* Kuwayama carrying *Candidatus* Liberibacter asiaticus. All correlations were significantly different from zero *p* < 0.0001.

		Female		Male	
		Brown	Green	Brown	Green
N		193	187	191	130
Female	Brown		0.848	0.942	0.808
	Green			0.861	0.786
Male	Brown				0.816

## Data Availability

The data are available in the Harvard Dataverse at https://dataverse.harvard.edu/dataset.xhtml?persistentId=doi:10.7910/DVN/6VMESN (accessed on 9 September 2023). Weather data are available at https://fawn.ifas.ufl.edu/ (accessed on 9 September 2023).
